# Latent Microsporidial Infection in Immunocompetent Individuals – A Longitudinal Study

**DOI:** 10.1371/journal.pntd.0001162

**Published:** 2011-05-24

**Authors:** Bohumil Sak, Martin Kváč, Zuzana Kučerová, Dana Květoňová, Kamila Saková

**Affiliations:** 1 Biology Centre of the Academy of Sciences of the Czech Republic, v.v.i., Institute of Parasitology, České Budějovice, Czech Republic; 2 Faculty of Agriculture, University of South Bohemia in České Budějovice, České Budějovice, Czech Republic; 3 Centers for Disease Control and Prevention, Atlanta, Georgia, United States of America; 4 Laboratory of Virology, České Budějovice Hospital, České Budějovice, Czech Republic; The George Washington University Medical Center, United States of America

## Abstract

**Background:**

Microsporidia (Fungi) have been repeatedly identified as the cause of opportunistic infections predominantly in immunodeficient individuals such as AIDS patients. However, the global epidemiology of human microsporidiosis is poorly understood and the ability of microsporidia to survive and multiply in immunocompetent hosts remains unsolved.

**Aims:**

To determine the presence of latent microsporidia infections in apparently healthy humans in the Czech Republic, the authors tested sera, urine and stool originating from fifteen persons within a three month period examined on a weekly basis.

**Methods:**

Sera, stool and urine samples originating from fifteen HIV-negative people at risk with occupational exposure to animals, aged 22–56 years, living in the Czech Republic were tested by indirect immunofluorescence assay (IFA) for the presence of specific anti-microsporidial antibodies, standard Calcofluor M2R staining for the detection of microsporidian spores in all urine sediments and stool smears and molecular methods for the microsporidial species determination.

**Results:**

Specific anti-microsporidial antibodies were detected in fourteen individuals, asymptomatic *Encephalitozoon* spp. infection was found in thirteen and *E. bieneusi* infection was detected in seven of those examined. While *E. hellem* 1A and *E. cuniculi* II were the major causative agents identified, seven different genotypes of *E. bieneusi* were recorded.

**Conclusions:**

These findings clearly show that exposure to microsporidia is common and chronic microsporidiosis is not linked to any clinical manifestation in healthy population. Moreover, our results indicate much higher incidence of microsporidial infections among an apparently healthy population than previously reported. These results open the question about the potential risk of reactivation of latent microsporidiosis in cases of immunosupression causing life-threatening disease.

## Author Summary

Microsporidia are a group of obligate intracellular parasitic fungi that have risen over the past two decades from obscure organisms to well recognized human pathogens. Out of 14 species reported to infect humans and causing more severe symptoms in immunocompromised individuals, microsporidia of the species *Encephalitozoon* and *Enterocytozoon bieneusi* are the most frequent causes of life-threatening chronic diarrhea and systemic disease in HIV patients and acute, self-limited diarrhea in immunocompetent persons. Although the diagnosis and clinical management of microsporidiosis cases have improved significantly recently, the epidemiology of human microsporidiosis is still unclear. To identify the occurrence of latent microsporidia infections in apparently healthy people, the authors tested sera, urine and stool originating from fifteen persons within a three month period. They found specific antibodies against microsporidia in sera originating from fourteen individuals, and using molecular tools, they detected microsporidial infection intermittently in all tested people. The presence of detectable amounts of microsporidial spores demonstrated that exposure to microsporidia is more common than previously believed and microsporidiosis is not linked to any clinical manifestations in healthy people. This finding should make the clinician more aware of the risk of this unapparent infection, its potential reactivation after immunosupression and consequences leading to life-threatening disease.

## Introduction

Microsporidia have emerged as causative agents of opportunistic infections in AIDS patients and other immunodeficient individuals. Several species of microsporidia can cause disease in humans. Intestinal microsporidiosis due to *Encephalitozoon* (*Septata*) *intestinalis* and *Enterocytozoon bieneusi* are most frequently reported among immunocompromised people including patients with acquired immune deficiency syndrome (AIDS) [1], [2] and other immunocompromised patients such as transplant recipients [3]–[6]. *Encephalitozoon cuniculi* and *E. hellem* are less prevalent among immunodeficient patients [7], [8]. Infections with microsporidia in immunocompetent individuals such as travelers have also been described [9], [10].

Although the most common clinical symptoms related to encephalitozoonosis among immunodeficient patients are chronic diarrhea and malabsorption, they can also cause systemic diseases. While immunocompetent persons often have mild or self-limiting disease, AIDS patients can experience weight loss and increased mortality [11].

Since the studies examining the prevalence of microsporidiosis have been limited to patients who are infected with human immunodeficiency virus (HIV) or who have AIDS, the global epidemiology of human microsporidiosis is poorly understood. Variation of spore shedding intensity of microsporidia was shown in both human and animals [12]–[16]. However, to our knowledge there have been no reports on the spore shedding pattern of microsporidia in immunocompetent humans. Therefore we aimed to study the pattern of microsporidia spore shedding in a cohort of asymptomatic apparently healthy people.

## Materials and Methods

### Ethics statement

The study was approved by the Hospital České Budějovice ethics committee (protocol no. 202/07).

Written informed consent was obtained from every person prior to examination.

### Stool samples

Between September and December 2007, a total of 180 individual stool and 180 urine samples were collected on the weekly basis for 3 months from fifteen HIV-negative people at risk of occupational exposure to various animals, such as farm ruminants, pigs, poultry and rodents. The male to female ratio was 8 (53%) to 7 (47%) with mean age of 35±11 years and range between 22–56 years. The samples were stored at 4°C in the dark without any conservation and examined immediately. Every specimen in the study was supplemented with data on the person's clinical symptoms (e.g., indigestion, abdominal pain).

### Serological examination

Prior to the study, serum samples were obtained from all individuals included and the presence of specific anti-microsporidial immunoglobulin G was tested by indirect immunofluorescence assay (IFA). IFA was performed with purified whole spores of *E. hellem*, *E. cuniculi* or *E. intestinalis* grown *in vitro* in VERO E6 cells and semi-purified spores of *E. bieneusi* at the concentration 10^5^ spores/well (spores kindly provided by Dr. G.S. Visvesvara, CDC Atlanta, GA, USA). Sera were serially diluted (1∶8, 1∶16, 1∶32, 1∶64, 1∶128 and 1∶256) in PBS and compared with negative and positive control sera. Sera with positive fluorescence at titers greater than 128 were considered positive.

### Microscopic examination

Standard Calcofluor M2R staining [17] was used for the detection of microsporidian spores in all urine sediments and stool smears. Stained slides were examined by fluorescence microscopy using UV light with a wavelength of 490 nm and at a magnification of 1000×. Positive control slides were used for each examination.

### Examination by molecular methods

The DNA was isolated from the stool and urine samples using homogenization by bead disruption using FastPrep–24 Instrument (MP Biomedicals, CA, USA) and DNA was extracted using commercially available isolation kit QIAamp DNA Stool Mini Kit (QIAGEN, Hilden, Germany) according to the manufacturer's instructions. Acquired DNA was stored at −20°C.

The nested PCR protocol by Katzwinkel-Wladarsch et al [18] amplifying the ITS region of *Encephalitozoon* spp. and *Enterocytozoon bieneusi* using microsporidia-specific primers was performed as described elsewhere [16]. As positive controls the following were used: DNA obtained from spores of *E. intestinalis* originally isolated from AIDS patients [19] and grown in *vitro* in VERO E6 cells in the Laboratory of Veterinary and Medical Protistology at the Institute of Parasitology ASCR, and DNA from spores of *E. bieneusi* of genotype D originally isolated from a pig [16]. PCR products were visualized on a 2% agarose gel containing 0.2 µg/ml ethidium bromide and directly sequenced on the ABI3730XL sequence analyzer (Applied Biosystems, Foster City, CA). Sequences were aligned and completed using programs ChromasPro (Technelysium, Pty, Ltd.) BioEdit and Clustal X 2.0.6 and compared with sequences in GenBank.

## Results

Specific anti-microsporidial antibodies were detected in fourteen out of fifteen tested people; 87% of sera reacted with *E. cuniculi*, 47% with *E. hellem*, 13% with *E. bieneusi* and none with *E. intestinalis* (Table 1). None of the individuals demonstrated any clinical symptoms (loose stool, indigestion, etc.).

**Table 1:** Seropositivity to *Enterocytozoon bieneusi* and *Encephalitozoon* spp. at the beginning of the tested period.

Personal identification number/control sera	Seropositivity – maximum titer			
	*E. hellem*	*E. intestinalis*	*E. cuniculi*	*Enterocytozoon bieneusi*
1	1∶64	1∶64	**1∶128**	1∶64
2	**1∶128**	1∶32	**1∶128**	1∶64
3	1∶64	1∶64	**1∶128**	1∶64
4	1∶64	1∶32	**1∶128**	1∶64
5	**1∶128**	1∶16	**1∶128**	1∶64
6	1∶32	1∶16	**1∶128**	1∶32
7	**1∶128**	1∶32	**1∶128**	1∶32
8	**1∶128**	1∶64	**1∶128**	1∶32
9	**1∶128**	1∶64	**1∶128**	1∶64
10	1∶32	1∶16	1∶32	**1∶128**
11	1∶32	1∶16	**1∶128**	1∶32
12	**1∶128**	1∶16	**1∶128**	**1∶128**
13	1∶32	1∶32	**1∶128**	1∶32
14	1∶64	1∶16	1∶64	1∶32
15	**1∶128**	1∶32	**1∶128**	1∶64
Negative serum 1	1∶8	1∶16	1∶32	1∶8
Negative serum 2	1∶16	1∶16	1∶16	1∶32
Negative serum 3	1∶8	1∶32	1∶32	1∶16
Positive serum 1	**1∶256**	1∶32	1∶16	1∶32
Positive serum 2	1∶32	**1∶128**	1∶64	1∶32
Positive serum 3	1∶32	**1∶128**	**1∶256**	1∶32
Positive serum 4	1∶8	1∶32	1∶32	**1∶128**

While no positive finding was revealed among samples of urine using microscopy, four stool samples originating from 3 persons were positive for microsporidia spores, which were subsequently molecularly characterized as *E. bieneusi*.

**Figure 1:**
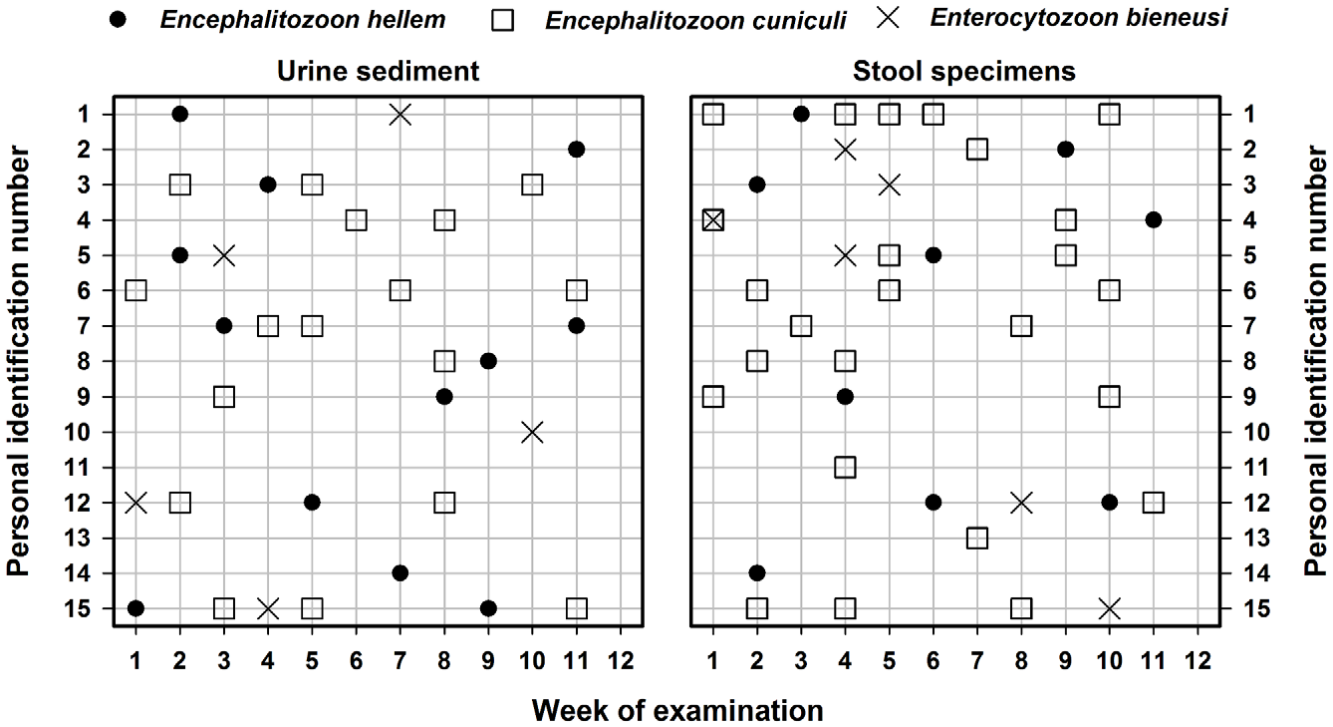
Frequency of microsporidia excretion by naturally infected immunocompetent humans. **A** urine sediments; **B** stool samples.

During the twelve week long observation of spore excretion, microsporidia were molecularly detected in 34 urine samples (19%) and 39 stool samples (22%) originating from all fifteen tested people. Each of the person excreted microsporidial spores intermittently in irregular intervals (Figure 1). The concurrent infection with two species of *Encephalitozoon*, *E. cuniculi* and *E. hellem*, and *Enterocytozoon bieneusi* was detected in 7 individuals, co-infection with *E. cuniculi* and *E. hellem* in three cases and monoinfections with *E. bieneusi*, *E. hellem* or *E. cuniculi* in one or three individuals, respectively. No *E. intestinalis* infection was detected. Whereas both *Encephalitozoon* spp. infections were more often found in urine, *E. bieneusi* was detected equally in urine and stool samples (Figure 1). While *E. hellem* or *E. cuniculi* infection were caused mainly by predominant genotype 1A or II, seven different genotypes of *E. bieneusi* including novel genotypes CZ4–CZ6 were identified in both urine and stool samples. All other ITS sequences from the study samples were a 100% match to the reference genotypes from GenBank listed in Table 2.

**Table 2:** Detection of microsporidia in urine and stool samples of naturally infected immunocompetent humans.

Species	genotype	Number of positive person	Number of positive specimens		Reference sequence
			urine	stool	
***E. hellem***		1	2	0	AY171241
	1A	10	10	9	AF338367
***E. cuniculi***	I	1	1	0	AF338410
	II	13	16	25	GQ422153
***E. bieneusi***	BfRmr2	1	1	1	EU849132
	H	1	1	1	AF135835
	EpbC	1	0	1	AF076042
	H/F	1	1	1	EU849130
	CZ4	1	2	0	HM143725
	CZ5	1	0	1	HM143726
	CZ6	1	0	1	HM143727

## Discussion

The actual extent of microsporidian infections is unknown. Microsporidia were, and still are, often overlooked and misdiagnosed because they are not specifically searched for in most diagnostic labs, they are rather small, and their staining with hematoxylin and eosin is not sufficient. Most of what is now known about human microsporidiosis can be attributed to the experience with patients infected with HIV [1], [2]. However, with increased awareness and improved diagnostics, microsporidia have become more frequently reported also in immunocompetent individuals, producing asymptomatic infections [20]–[22]. Despite limited sample number our findings showed a well-supported correlation between spore presence in excretions and seropositivity, which discriminates the actual latent microsporidiosis from simple consumption and passage of spores through the intestinal tract.

Intermittent spore shedding for a long period has been experimentally demonstrated for several hosts including rabbits with *E. cuniculi* [12], wild-type mice with *E. intestinalis* [13], pigs with *E. bieneusi* [15], budgerigars with naturally acquired *Encephalitozoon* spp. infection [23] and HIV-positive patients with *E. bieneusi* [14]. The persistence of microsporidia despite resolution of the intestinal disorder suggests that microsporidia infection may cause clinical symptoms (e.g., diarrhea) during the early stages of infection that could be overlooked and resolved even though the microsporidia persist.

Our survey was performed on a limited sample size from a highly selected population, which could result in decreased statistical power. On the basis of present results it is obvious, that prevalence data of microsporidial infection reported by various authors reaching up to 38% the case of *Encephalitozoon* spp. and 51% for *E. bieneusi*, could be hampered by collection of only a single sample for diagnosis, especially in low level infections. While the twelve week sampling enabled us to detect *E. cuniculi* in 86% of tested people, *E. hellem* in 66% and *E. bieneusi* in 47%, the hypothetical individual single sampling performed at any day would identify *E. cuniculi* in only 0–27% of persons, *E. hellem* in 0–13%, and *E. bieneusi* in 0–13%.

Based on data in the literature and our experience, it seems that the incidence of microsporidial infections is much higher than previously reported and microsporidia may represent neglected etiological agent of more common diseases. However, it is not known how extensive such silent infections are in asymptomatic carriers, including both humans and animals, which have been reported increasingly to harbour various species and genotypes of microsporidia [16], [24], [25]. Moreover, the fact that microsporidia DNA were detected in urine sediments suggests, that microsporidia are able to disseminate also in immunocompetent hosts despite previously reported protective T-cell mediated adaptive immunity together with several components of innate immunity [26], [27]. Furthermore, the majority of prevalence studies currently rely on detection of spores in stool samples only. The results of this study clearly showed that infected seropositive person could excrete detectable amount of microsporidial DNA via urine, nevertheless examination of stool sample will be negative. Detection of specific antibodies seems to be more sensitive than one-shot detection of spores and can provide more accurate information about ongoing microsporidia infection.

In conclusion, studies focusing on the epidemiology of microsporidiosis will more clearly define the environmental sources of microsporidia that pose a risk for transmission so that preventative strategies can be implemented. Since no data exist about latent infection in immunocompetent carriers, possible infection reactivation in these individuals and person to person transmission risk via organ donation, such epidemiological data must be compared with experiments that could solve this question definitively. Moreover, using detection methods with a high sensitivity, such as PCR, and consecutive sampling from every individual is recommended to provide more precise epidemiological data.

## Supporting Information

Checklist S1 STROBE checklist.
(DOC)
