# Supplementary material for: Latent Microsporidial Infection in Immunocompetent Individuals – A Longitudinal Study
Source: PLoS Negl Trop Dis. 2011 May 24;5(5):e1162. doi: 10.1371/journal.pntd.0001162 (PMC3101169; doi:10.1371/journal.pntd.0001162)
Supplement: Checklist S1 — STROBE checklist. (DOC) [file pntd.0001162.s001.doc]

STROBE Statement—Checklist of items that should be included in reports of ***cross-sectional studies***

|  | Item No | Recommendation |
| --- | --- | --- |
| **Title and abstract** | X | (*a*) Indicate the study’s design with a commonly used term in the title or the abstract |
|  |  | (*b*) Provide in the abstract an informative and balanced summary of what was done and what was found |
| Introduction | | |
| Background/rationale | X | 1^st^ – 2^nd^ paragraph |
| Objectives | X | 3^rd^ paragraph |
| Methods | | |
| Study design | N/A | Present key elements of study design early in the paper |
| Setting | X | 2^nd^ section |
| Participants | N/A | (*a*) Give the eligibility criteria, and the sources and methods of selection of participants |
| Variables | N/A | Clearly define all outcomes, exposures, predictors, potential confounders, and effect modifiers. Give diagnostic criteria, if applicable |
| Data sources/ measurement | X | 3^rd^ – 5^th^ section |
| Bias | N/A | Describe any efforts to address potential sources of bias |
| Study size | N/A | Explain how the study size was arrived at |
| Quantitative variables | N/A | Explain how quantitative variables were handled in the analyses. If applicable, describe which groupings were chosen and why |
| Statistical methods | N/A | (*a*) Describe all statistical methods, including those used to control for confounding |
|  |  | (*b*) Describe any methods used to examine subgroups and interactions |
|  |  | (*c*) Explain how missing data were addressed |
|  |  | (*d*) If applicable, describe analytical methods taking account of sampling strategy |
|  |  | (*e*) Describe any sensitivity analyses |
| Results | | |
| Participants | X | 1^st^ paragraph |
|  |  | (b) Give reasons for non-participation at each stage |
|  |  | (c) Consider use of a flow diagram |
| Descriptive data | N/A | (a) Give characteristics of study participants (eg demographic, clinical, social) and information on exposures and potential confounders |
|  |  | (b) Indicate number of participants with missing data for each variable of interest |
| Outcome data | X | 1^st^ – 3^rd^ paragraph |
| Main results | X | 3^rd^ paragraph |
|  |  | (*b*) Report category boundaries when continuous variables were categorized |
|  |  | (*c*) If relevant, consider translating estimates of relative risk into absolute risk for a meaningful time period |
| Other analyses | N/A | Report other analyses done—eg analyses of subgroups and interactions, and sensitivity analyses |
| Discussion | | |
| Key results | X | 1^st^ – 3^rd^ paragraph |
| Limitations | X | 3^rd^ paragraph |
| Interpretation | X | 4^th^ paragraph |
| Generalisability | X | 5^th^ paragraph |
| Other information | | |
| Funding | X | 1^st^ paragraph of Funding |

*Give information separately for exposed and unexposed groups.

**Note:** An Explanation and Elaboration article discusses each checklist item and gives methodological background and published examples of transparent reporting. The STROBE checklist is best used in conjunction with this article (freely available on the Web sites of PLoS Medicine at http://www.plosmedicine.org/, Annals of Internal Medicine at http://www.annals.org/, and Epidemiology at http://www.epidem.com/). Information on the STROBE Initiative is available at www.strobe-statement.org.
